# Post-Cracking Properties of Concrete Reinforced with Polypropylene Fibers through the Barcelona Test

**DOI:** 10.3390/polym15183718

**Published:** 2023-09-10

**Authors:** Alexandre Almeida Del Savio, Darwin La Torre Esquivel, Joaquín M. García Landeo

**Affiliations:** Civil Engineering Department, Universidad de Lima, Lima 15023, Peru; dalatorr@ulima.edu.pe (D.L.T.E.); joagarlan@gmail.com (J.M.G.L.)

**Keywords:** polypropylene fiber, post-cracking behavior in bending, toughness, residual strength, Barcelona test, UNE 83515, fiber-reinforced concrete

## Abstract

The Barcelona method was developed as an alternative to other tests for assessing the post-cracking behavior of fiber-reinforced concrete, with the main advantage being that it uses significantly smaller specimens compared to other methods. For this reason, it can provide a solution for characterizing concrete in hard-to-reach constructions such as roads and tunnels. On the other hand, polypropylene (PP) fibers have gained increased attention in recent years within the scientific community due to their high tensile strength and cost-effectiveness. This research aimed to understand the influence of PP fiber volume, slenderness (l/d), and reinforcement index on post-cracking properties of concrete, including toughness and residual strength (f_res), using the Barcelona method. Three fiber volumes, 0.4%, 0.8%, and 1.2%, and three slenderness ratios, 46.5, 58.1, and 69.8, were employed in normal-strength concrete. In addition to the reference mixture without fibers, 10 mixtures were prepared with 10 specimens each, resulting in a total of 100 specimens. Pearson’s hypothesis test was employed to determine the existence of correlations between variables, followed by scatter plots to generate predictive equations between post-cracking properties and fiber attributes. The results indicated no direct correlation between fiber slenderness and post-cracking properties. Regarding fiber volume, there was a correlation with residual strength but not with toughness. However, the combined effect of volume and slenderness, the reinforcement index, correlates with the post-cracking properties of concrete. Finally, four predictive equations for toughness and residual strength were derived based on the reinforcement index. These equations can prove valuable for designing structures made of polypropylene fiber-reinforced concrete.

## 1. Introduction

In the realm of contemporary construction, plain concrete stands out as an essential material due to its simplicity and versatility, being used in various structures and projects. However, the increasing demands for structural strength and durability have revealed plain concrete’s limitations under intense stress and load, leading to the emergence of fiber-reinforced concrete (FRC). Compared with plain concrete, FRC is known for its enhanced residual strengths and toughness [1,2]. The FRC is widely applied in constructing subway tunnels, industrial floors, rail bases, machine foundations, and underground mining [3]. The energy absorption capacity, or toughness, is an important characteristic of structures subjected to seismic, fatigue, impact, and blast loads [4]. The toughness of a specimen is equal to the area under its load-deflection curve up to a certain displacement [5]. Its magnitude depends directly on the geometry of the test specimen and the type of fibers [6].

The most used fibers are steel and polypropylene fibers. PP fibers are synthetic fibers made from isotactic polypropylene polymerized with propylene [7]. This type of fiber has a high melting point (165 °C) and good corrosion resistance. Due to their hydrophobicity, polypropylene fibers can be evenly distributed in concrete [8]. In addition, they have a greater effect on shrinkage reduction and decrease the spalling effect in high-strength FRC subjected to high temperatures [9]. Del Savio et al. (2022) [10] found that PP fibers with lengths of 50 and 60 mm increase 34% and 35%, respectively, in the tensile strength when the dosage is at 1.20%. Also, PP fibers with lengths of 60 mm show an increase of 3.80, 8.46, and 12.09% when the dosage is 0.4, 0.8, and 1.2%, respectively, in the modulus of rupture.

On the other hand, since the decade of 2010, the Barcelona method has been developed to evaluate toughness, and the test configuration consists of double punching a cylinder with a slenderness equal to unity. This method is more economical than other analogous methods due to the small dimensions of the specimen, and several authors mention that it has a lower coefficient of variation (COV) than other methods [6,11,12,13,14]. However, most of these studies work with steel fibers. Therefore, it is important to know the technical and statistical reliability of the Barcelona method as a decision-making tool when choosing the most appropriate method.

Mechanical tests to obtain the post-cracking behavior of FRC are part of the quality control at construction sites. The three-point bending test (3 PB), established in the European Standard EN 14651 [15], specifies a method for determining the flexural tensile strength of concrete with metallic fibers. This method calculates the limit of proportionality (LOP) and residual strength values. The four-point bending test (4 PB), specified in ASTM C1609 [16], evaluates the flexural behavior of FRC using parameters derived from the load-deflection curve obtained by testing a supported beam under loads applied. It lets us know the cracking load, residual strengths, and toughness. However, these tests are characterized by their high dispersion of results because the beams present a small fracture surface [11].

Moreover, the properties depend directly on the specific number of fibers in the cracked section [12]. Alternatively, EN 14488 [17] and ASTM C1550 [18] allow calculating the flexural toughness of FRC through square and circular panels subjected to a central point load. The double punch test (DPT) of Molins et al. [19] is an alternative to evaluate the post-cracking behavior of FRC. This “Barcelona method” test presents less dispersion with a COV of results below 13% [20]. The standard developed for this test is UNE 83 515 [21]. This method allows for determining FRC’s cracking resistance, toughness, and residual tensile strength. In addition, it has proven to be suitable for controlling FRC on-site. Unlike the 3 PB and 4 PB flexural tests, the double punching test is characterized by using cylindrical specimens with larger cracking surfaces [22].

In general, it is known that the higher the reinforcement index (RI), the higher the toughness of concrete [23,24]. Carmona et al. [25] studied six FRC mixtures with different amounts of steel fibers and plastic fibers to measure the circumferential and axial displacement of the specimens during the tests. The results showed that as the fiber content increases, the results present a higher dispersion in the range of 4.78 to 14.35%. Carmona et al. [12] introduced the concept of toughness index to quantify the post-cracking behavior of FRC with steel fibers, with results showing a dispersion between 5.56 and 14.41%. Aire et al. [26] conducted a comparative study applying the Barcelona test between direct tensile strength and toughness results in 100 × 150 mm molded specimens and 93 × 153 mm control specimens of FRC with steel fibers. The dispersion of their results measured at 4 mm circumferential elongation varied in the range of 13.2 to 35.7%. Molins et al. [19] studied the influence of fiber addition on the toughness of concrete. Their results indicated variability ranges of 13.1 to 22.5%.

Investigations about the post-cracking properties of normal-strength concrete with polypropylene fibers are limited. Del Savio et al. (2023) [27] concluded with a strong correlation between fiber parameters and post-cracking properties with a Pearson’s correlation coefficient, r of 0.90. Molins et al. [19] demonstrated a direct correlation between toughness calculated using the Barcelona method and the beam flexural method. Choumanidis [3], using the Barcelona method, found that polypropylene fibers perform better when the crack width is larger than steel fibers. This conclusion was supported by comparing the slopes of the total circumferential opening displacement (TCOD) curve versus toughness for crack widths of 0.5 mm and 3.5 mm, respectively; the percentage difference in slopes increased from 10% to 41% with a larger crack width. Carmona et al. [22] found that more synthetic fibers lead to greater toughness when testing concrete specimens with 4, 8, and 12 kg/m^3^ of synthetic fibers. The influence of slenderness and RI on residual strength and toughness of concrete calculated through the Barcelona method has not been reported, and it is important to expand the understanding beyond fiber volume to consider the influence of the RI. In a separate study, Del Savio (2022) [28] investigated the correlation between the RI and the mechanical properties of concrete. This investigation employed two methods: Ultrasonic Pulse Tests (UPV) and dynamic elasticity modulus. Additionally, developing predictive equations for statistically correlated relationships is essential.

The search for a simple, fast method with reliable results is fundamental to establishing adequate quality control of the post-cracking behavior of polypropylene fiber-reinforced concrete (PPFRC). In this study, the post-cracking behavior of FRC, toughness, and residual strength were evaluated through the DPT, also known as the “Barcelona method,” by varying polypropylene fiber properties, length (l), volume (V_f), and the RI. Then, a statistical analysis was made to study the correlations of independent variables (IV) with the post-cracking properties, which consisted first of elaborating a total of 10 specimens per concrete mixture, resulting in the testing of 100 specimens. Following this, hypothesis tests were performed to determine each correlation’s significance value (*p*). In addition, the coefficient of toughness variation at different casting locations and with different types of fibers used in the fiber-reinforced concrete was evaluated using previous research results. Finally, a predictive equation was developed to calculate toughness and residual strength as a function of the RI. The experimental plan was developed using three dosages of polypropylene fibers with values corresponding to 0.4, 0.8, and 1.2%. Three fiber lengths were also used: 40, 50, and 60 mm. The test was performed according to the UNE 83515 standard developed in Spain by AENOR [21]. This research is organized in the following order: in Section 1, the introduction is presented. Section 2 presents the characteristics of concrete, including aggregate properties, mix design, and experimental procedure. It also includes the definition of the hypothesis test. The execution of the experimental test is described in Section 2. Section 3 presents the discussion and analysis of the results and the formulation of a predictive equation for toughness based on the FRC experimental results. Finally, the conclusions are specified in Section 4.

## 2. Materials and Methods

### 2.1. Research Design

Fiber-reinforced concrete was made with type I Portland cement according to ASTM C150 [29]. This cement is characterized by being general purpose, having a high heat of hydration, and rapid strength development.

**Figure 1:**
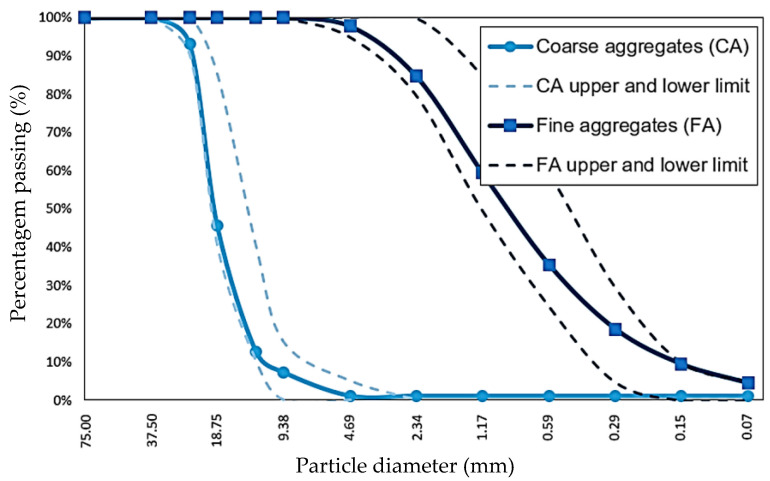
Aggregate grain size curves.

The coarse aggregate (CA) used was crushed stone. According to their gradation, the coarse aggregates comply with HUSO N°56 according to ASTM C33 [30]. The CA had a maximum nominal size of 25.4 mm. The sand used was river sand and met the particle size requirements of ASTM C33 standard. The fineness modulus of the natural river fine aggregate was 2.95. The particle size curve of the aggregates is shown in Figure 1. 

The PP fibers used had a density of 910 kg/m^3^, a modulus of elasticity (E) of 4.70 GPa, and a tensile strength (f_t) of 540 MPa. The length of the fibers was variable, 40, 50, and 60 mm. All fibers had the same diameter of 0.86 mm.

A high-range water-reducing and retarding additive type G, according to ASTM C494 [31], with a density of 1200 kg/m^3^, was used.

### 2.2. Specimen

The concrete was designed to achieve a strength of 40 MPa and a slump of 100 mm. The design method used was the ACI [32]. The water–cement ratio was 0.45. The high-range water-reducing admixture was placed at a dosage of 1.40% by weight of the cement. Fibers were placed in three dosages (0.4, 0.8, and 1.2%) and three lengths (40, 50 and 60 mm). The mixed designs are shown in Table 1.

### 2.3. Barcelona Test

**Figure 2:**
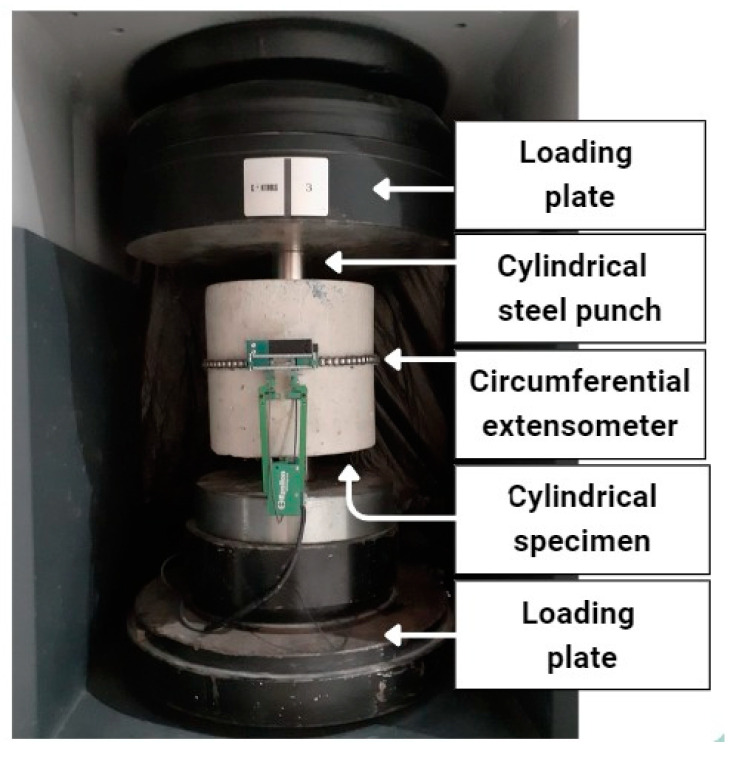
Test setup according to UNE 83515 [21].

The Barcelona method was performed according to the UNE 83515 standard developed in Spain by AENOR [21]. The objective of the test was to determine the toughness of the concrete reinforced with fibers, measured in Joules (J). The test was performed on cylindrical specimens with a diameter of 150 mm and a height of 150 mm. The Barcelona method consists of subjecting the FRC to a double punch test employing two cylindrical steel punches, with a height of 24 mm and a diameter of 37.5 mm, centered on the lower and upper surfaces of the specimens. A circumferential extensometer is placed at the mid-height of the specimen using a high-precision chain instrument. This instrument allows measurement of the total circumferential opening displacement (TCOD) connected to a clip-gauge. The press piston applies a constant axial displacement rate of 0.5 mm/min. The test ends when the axial displacement is at least 6 mm. Figure 2 shows the laboratory test setup.

The indirect tensile strength of the Barcelona test was determined through the formulation proposed by UNE 83515 in Equation (1).

**Figure 3:**
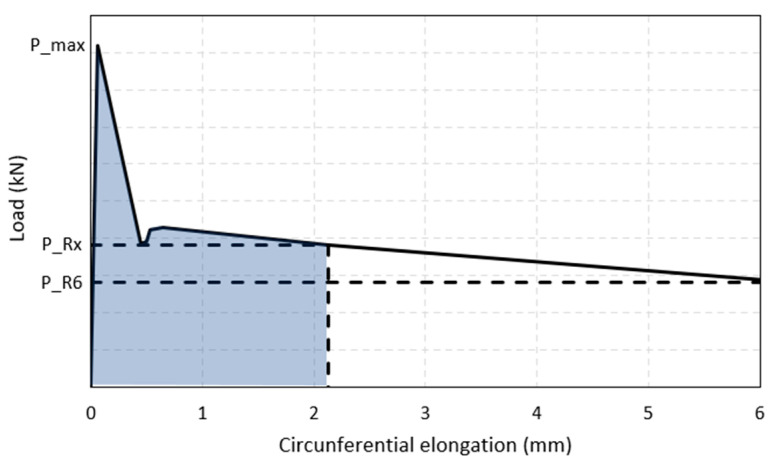
Resulting curve according to UNE 83515 [21].

(1) f_ct=(4 P_max)/(9 π a H)

where P_max (N) is the maximum load that produces cracking, a (mm) is the diameter of the load application disk, and H (mm) is the height of the specimen. Figure 3 represents the curve resulting from the Barcelona test, where P_RX is the residual load for a TCOD of RX mm, P_R6 is the residual load for a TCOD of 6 mm.

A total of 10 cylindrical specimens were made for each mixture for further statistical support. The methodology proposed by Gill et al. for rock masses [33] was used to determine this quantity. One of the curves from each series was selected as the representative curve to visualize the shape of at least one of the resulting curves.

### 2.4. Mixing Procedure

**Figure 4:**
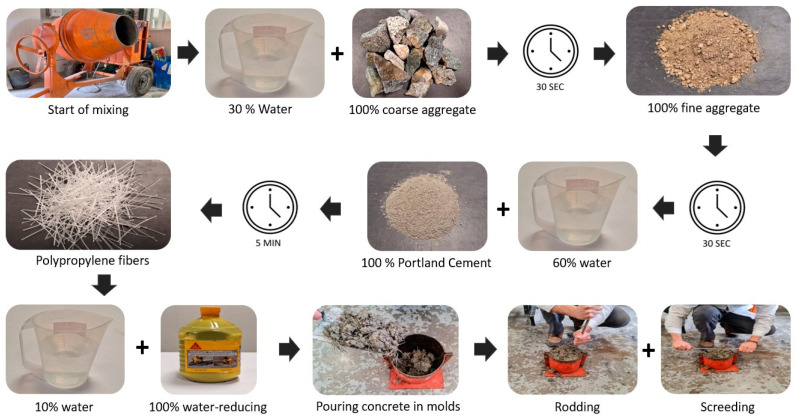
Specimen-manufacturing process.

The mixing procedure was carried out in four stages. In the first stage, 30% of the total water was added along with 100% of the coarse aggregate and mixed for 30 s. In the second stage, 100% of the fine aggregate was added and mixed again for 30 s. In the third stage, 100% of the cement was added, and 60% of the total water was mixed for 1 min. In this stage, the fibers were added progressively until the mixing was finished. Finally, the remaining water was added with the high-range water-reducing additive and mixed for 5 min. The molds were filled in three equal layers and compacted by rodding for the fresh concrete slump tests. For pouring, the 150 mm × 150 mm metallic cylinders were filled with fiber concrete in three layers and compacted manually using a tamping rod. For the slump test and pouring, the layers were compacted 25 times using a tamping rod to ensure uniformity of the concrete. Shortly after that, screeding was performed with a screeding trowel. One day later, the samples were demolded and placed in an outdoor curing pool for 28 days. Figure 4 shows the concrete pouring sequence in metallic cylinders.

### 2.5. Hypothesis Test

Pearson’s hypothesis test is valuable for confirming the statistical significance of correlations between variables. The outcomes of Pearson’s test are *p*-value and R-value, which indicate the significance level and correlation coefficient (R), respectively. Together, these values quantify the correlation’s strength between variables. The *p*-value indicates the probability of being wrong. The significance level is expressed in terms of probability, and the most common values are 0.05 and 0.01 [34]. However, in previous studies, it has been found advantageous to set this value as 0.10 for materials exhibiting a high coefficient of variation [10,27,28,35].

Furthermore, the null hypothesis contradicts or denies what the research hypothesis affirms; in essence, it serves as the counterpart to the research hypothesis [34]. In this research, the null hypothesis can be rejected or not rejected according to the significance level results. If the *p*-value is less than 0.10, the null hypothesis is rejected. The null hypothesis is not rejected if the *p*-value is more than 0.10.

The R-value is the correlation coefficient of the linear regression. R-value between 0% and 5% means that the correlation does not exist; between 5% and 20% is very poor; between 20% and 40% is poor; between 40% and 60% is moderate; between 60% and 80% is considerable; between 80% and 95% is strong; between 95% to 99.9% is very strong; and a value of 100% is perfect.

## 3. Results and Analysis

The results of the FRC in fresh and hardened states and their respective COV are presented in Table 2. According to the data obtained from the hardened condition tests, it was found that the average value of compressive (f_c) and tensile strength (f_t) was 42.2 MPa and 5.6 MPa, respectively.

**Figure 5:**
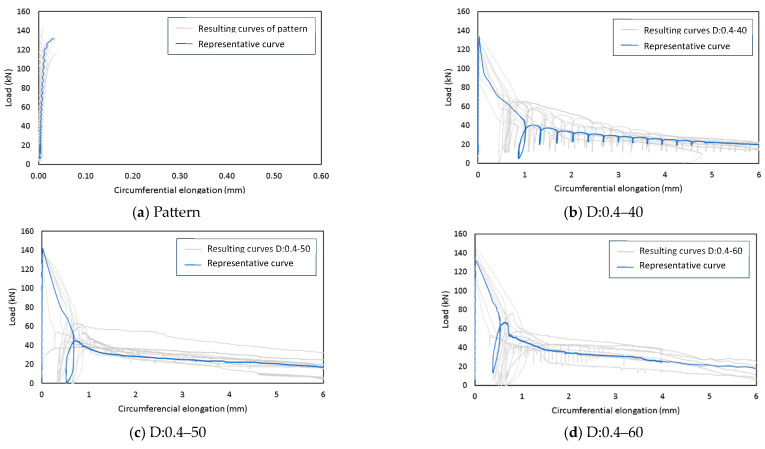

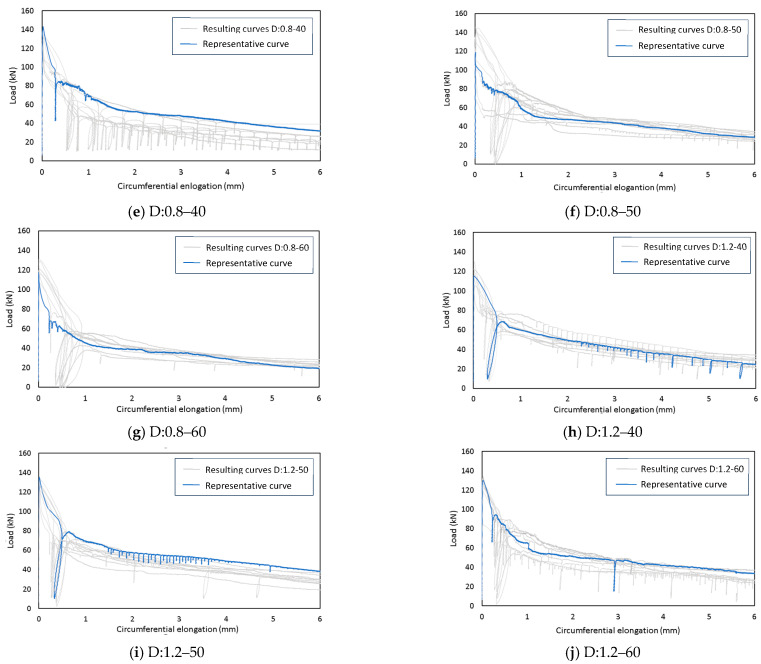
Circumferential load-deformation diagram in the specimens (**a**) pattern, (**b**) D:0.4–40, (**c**) D:0.4–50 (d), D:0.4–60, (**e**) D:0.8–40, (**f**) D:0.8–50, (**g**) D:0.8–60, (**h**) D:1.2–40, (**i**) D:1.2–50, (**j**) D:1.2–60.

Figure 5 shows the test’s Total Crack Opening Displacement (TCOD) diagram. The gray lines represent the resulting curves, and the blue lines symbolize the representative curves. In Figure 5a, a brittle fracture is observed corresponding to the pattern. In the case of FRC mixtures, they presented a similar behavior after fracture. A significant decrease in strength was noticeable, followed by a softening behavior in the curve. It is important to note that the strength of all mixtures appeared to converge around 140 kN. In Figure 5b, representing the mixture with the lowest RI, the strength initially decreased from 140 kN to 40 kN, corresponding to a 71% reduction in strength. Subsequently, a softening behavior in the curve decreased from 40 kN to 30 kN when 6 mm of elongation was reached. However, in Figure 5j, corresponding to the mixture with the highest RI, the strength decreased from 140 kN to 60 kN initially, representing an approximately 57% reduction. Furthermore, this sample presented a gradual softening behavior that reduced the strength from 60 kN to 40 kN when 6 mm of elongation was reached.

Table 3 shows the flexural mechanical properties of concrete reinforced with polypropylene fibers using the Barcelona test. For elastic properties, the maximum applied load (P_max) and the flexural tensile strength (f_ct) are shown. Regarding post-cracking properties, the results of residual load (P_4 mm, P_6 mm), residual strength (f_res_4 mm, f_res_6 mm), and toughness (T_4 mm, T_6 mm), all measured up to a deflection of 4 and 6 mm, respectively, are shown. The mixture with a 0.8% dosage and 50 mm of fiber length presented a Toughness, at 4 mm of elongation, of 227.1 N × m with a COV of 15.4%. Pujadas et al. [36] achieved a Toughness, at 4 mm, of 265.8 N × m with a COV of 11.6% for a mixture with 0.99% dosage and a fiber length of 48 mm. Molins et al. [19] obtained a toughness of 233.8 N × m with a COV of 22.5% using a dosage of 0.71% and a fiber length of 48 mm. Carmona et al. [22] achieved a toughness of 230.0 N × m with a dosage of 0.88% and a fiber length of 54 mm.

### 3.1. Post-Cracking Properties

Table 4 shows the results of Pearson’s 35 hypothesis tests between the variables of the polypropylene fibers: slenderness (l/d), fiber volume (V_f), and reinforcement index (RI=V_f×l/d), and the post-cracking properties of the concrete: tensile strength, residual strength, and toughness. This analysis concluded that there was no correlation between fiber volume, slenderness, and reinforcement index in the tensile strength calculated using the Barcelona method because the *p*-value in all correlations, from 1 to 7, was greater than 0.100. The correlations with the residual strength calculated at 4 mm and 6 mm were specified in items 8 to 21. It was observed that there was a correlation between fiber volume and residual strength. However, the slenderness did not influence the residual strengths. There was a correlation with the reinforcement index in f_res_4 mm and f_res_6 mm. The correlations with toughness calculated at 4 mm and 6 mm TCOD are shown in items 22 to 35. The results showed no correlation between the fibers’ volume and the calculated toughness. On the other hand, there was a correlation between fiber slenderness and toughness, but only for low fiber volumes, 0.4%. Finally, there was a correlation between the reinforcement index and toughness in both cases, T_4 mm and T_6 mm.

#### 3.1.1. Toughness

**Figure 6:**
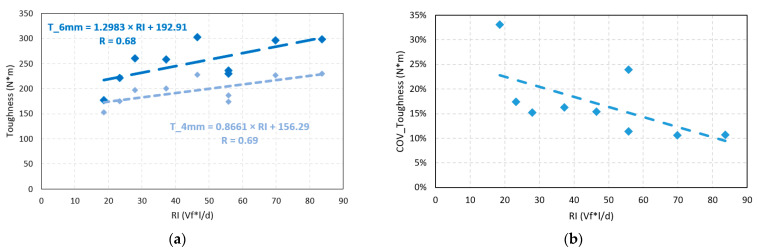
(**a**) FRC toughness as a function of RI, (**b**) coefficient of toughness variation as a function of RI.

Figure 6a shows the trend lines of the relation between toughness and fiber reinforcement index measured for 4 and 6 mm TCOD. The RI is the product of slenderness and fiber volume. The *p*-values of the correlation between toughness and RI for tests performed up to 4 and 6 mm strain were 0.039 and 0.044, respectively. It was observed that these correlations were significant since both values were less than 0.100. The correlation coefficients, R, were 0.69 and 0.68, respectively, indicating considerable correlations. Finally, the trend line showed that an increase of 10 RI units will increase 12.98 J for T_6 mm and 8.86 J for T_4 mm. Figure 6b shows the relation between the coefficient of toughness variation with the strengthening index. From the analysis, the higher the RI, the lower the COV. This is supported by Pearson’s test, which resulted in a *p*-value of 0.041, rejecting the null hypothesis.

From the linear regressions of T_6 mm and T_4 mm with the RI, Equations (2) and (3), valid for synthetic fibers, are obtained:

(2) T_6 mm=1.298×RI+192.91

(3) T_4 mm=0.866×RI+156.29

These equations are useful to calculate the toughness as a function of the reinforcement index of polypropylene fibers for concrete with compressive strengths of 40 to 45 MPa, slump ranging from 46 to 240 mm, made with aggregates with a maximum nominal size of 25.4 mm.

#### 3.1.2. Residual Strength

**Figure 7:**
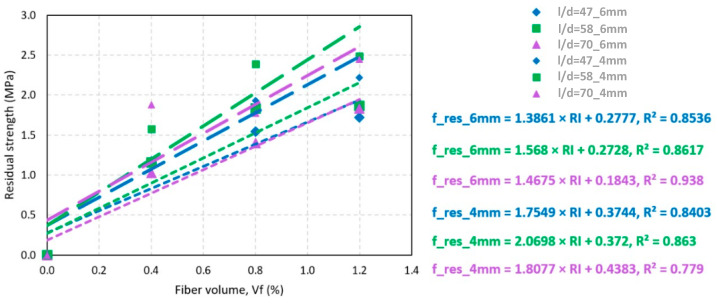
Residual strength as a function of fiber volume.

Linear regression allows predicting the behavior of the residual strength measured at TCOD of 4 and 6 mm from the dosage values in the fibers’ volume. Figure 7 shows the lines representing the trend of residual strength concerning fiber volume. For the residual strengths measured at 4 mm, the *p*-values of the correlations were 0.083, 0.071, and 0.117 for slenderness of 47, 58, and 70, respectively. For the residual strengths measured at 6 mm, the *p*-values were 0.077, 0.071, and 0.035, respectively. These correlations were statistically significant since five of the six values were less than 0.100. Consequently, the hypothesis that there is a correlation between variables was accepted.

**Figure 8:**
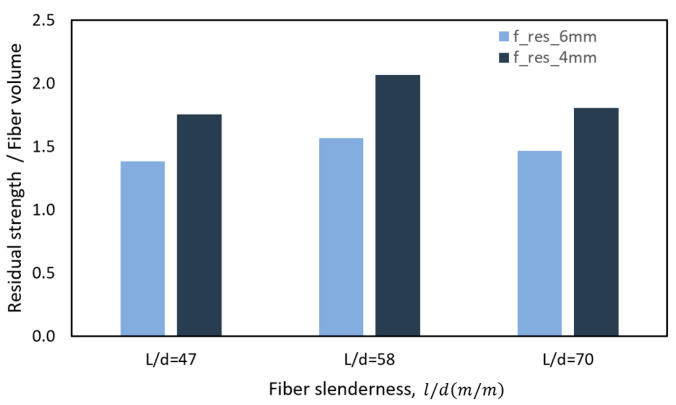
Increase in toughness per fiber volume as a function of fiber slenderness.

Figure 8 shows that the increase in residual strength due to the increase in fiber volume was independent of the fiber slenderness used, with an average value of 1.88 MPa for f_res_4 mm and 1.47 for f_res_6 mm. Finally, the maximum residual strength corresponding to f_res_4 mm was that of the D:1.2–50 blend with a value of 2.49 MPa; similarly, for f_res_6 mm, the maximum residual strength was that of the D:1.2–50 blend with a value of 1.9 MPa.

**Figure 9:**
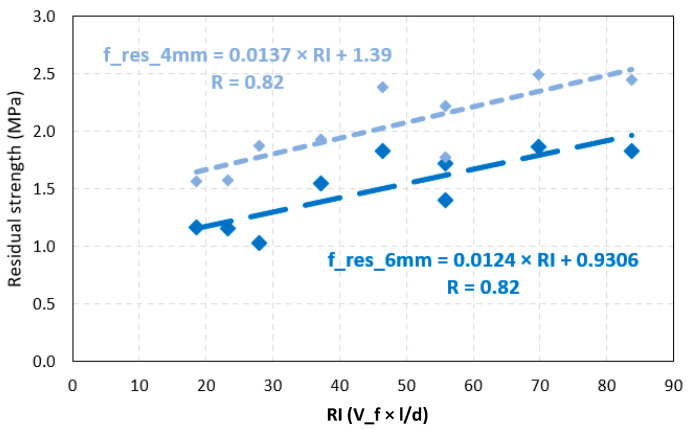
Linear regression of FRC residual strength as a function of fiber reinforcement index.

Figure 9 shows the relation between the residual strength of concrete and the fiber RI for 4 and 6 mm TCOD. The *p*-values for the correlations between residual strength and RI were 0.006 and 0.002. These figures are very close to zero. Therefore, the probability of error was low. The correlation coefficients, R, were 0.82 and 0.85 for the 4 and 6 mm TCOD, respectively. This shows strong correlations in both cases. Finally, the trend line shows that 10 RI units will increase by 0.124 for f_res_6 mm and 0.137 MPa for f_res_4 mm.

From the linear regressions of f_res_6 mm and f_res_4 mm with the RI, Equations (2) and (3) valid for synthetic fibers are obtained:

(4) f_res_4 mm=0.014×RI+1.390

(5) f_res_6 mm=0.012×RI+0.931

These equations are useful, as in toughness, to calculate the residual strength as a function of the RI of polypropylene fibers for concrete with compressive strength of 40 to 45 MPa, slump ranging from 46 to 240 mm, elaborated with aggregates with a maximum nominal size of 25.4 mm.

**Figure 10:**
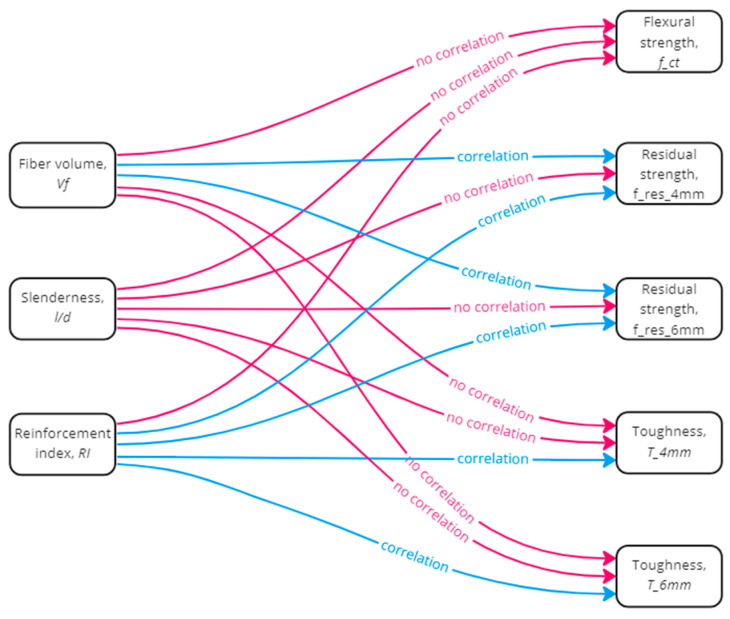
Correlation between the variables of synthetic fibers and post-cracking properties.

Figure 10 shows graphically the existence of a correlation between the variables studied. In general, it can be concluded that flexural strength is not determined by the placement of fibers in the mix; rather, it is determined by the concrete matrix. The residual strengths are influenced by the volume of fibers and by the RI. It may seem that the latter is a consequence of the former. Still, the significance level of the correlations with the RI was lower than in the case of the correlations with the volume of fibers. In other words, although the hypothesis that there is no correlation between fiber slenderness and residual strength cannot be rejected, slenderness does have an influence, although to a lesser degree. Finally, toughness was not influenced by fiber volume or slenderness when these were modified separately; but it was influenced by the combined effect of both, i.e., by the RI.

### 3.2. Comparative Analysis of Toughness as a Function of RI

**Figure 11:**
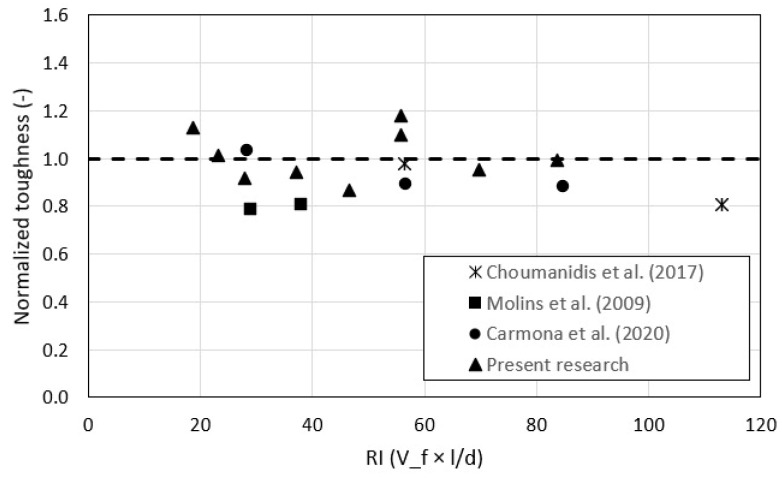
Calculated and normalized toughness values for different studies [3,19,22,36].

A compilation of results of toughness and residual strength calculated using the Barcelona method was made (see Table 5). The most studied fibers were steel and synthetic fibers. In addition, the place where the concrete was poured, in the laboratory and the underground mining, was also identified. The modulus of elasticity of the fibers (E), volume (V_f), length, diameter, slenderness (l/d), and reinforcement index (RI) were identified. In addition, the residual strength and toughness with their respective coefficients of variation were annotated. In Figure 11, one can observe the normalized toughness graph versus the RI. The developed empirical predictive Equations (2) and (3) were applied to the results of other authors collected in Table 5. In all cases, the results are underestimated. For Choumanidis et al.’s results [3], the values were underestimated by 11% compared to the experimental ones; for Molins’ results [19], by 20%; whereas for Carmona et al.’s results [22], by 6%.

### 3.3. Variance Analysis

**Figure 12:**
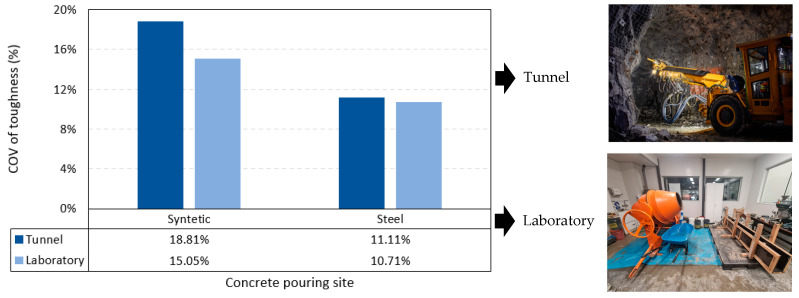
Coefficients of variation considering pouring site and fiber material.

The average coefficient of toughness variation of the results of this research and several other authors was 12.5%. It has been seen that the COV in specimens made in underground mining [6,22], 15.0%, is higher than the COV of specimens made in the laboratory [11,12,19,25,26,36,37], 12.9%. This is possible because concrete is not always of the same quality at different points of the structures; however, in the laboratory, all specimens are made under standardized and controlled conditions. On the other hand, the COV of specimens reinforced with synthetic fibers [11,19,22,36], 16.9%, is higher than the COV reinforced with steel fibers [12,25,26,36,37], 10.9%. The reason is that synthetic fibers contribute lower toughness to the concrete concerning steel fibers due to their lower modulus of elasticity. It has been seen that concretes with lower toughness have greater dispersion in the results. From the analysis of the data, it was also deduced that the higher the tenacity of the concrete, the lower the COV. The summary of this analysis is shown in Figure 12.

## 4. Conclusions

In this research, an extensive experimental campaign was carried out to statistically evaluate the results of the toughness and residual strength of polypropylene fiber-reinforced concrete calculated with the Barcelona method. For this purpose, fiber volume and slenderness were used as independent variables. The statistical evaluation consisted of (1) determining if there is a correlation between each independent variable with each of the post-cracking properties of PPFRC and generating empirical equations; and (2) comparing and calculating the COV of toughness and residual strength results with the results of other research.

- There was a correlation between fiber volume and residual strength, with a Pearson correlation coefficient of 0.82 showing a strong relationship. On the other hand, in this method, the null hypothesis that proposed no correlation between fiber volume and toughness cannot be rejected. However, there was a correlation between the reinforcement index with toughness and residual strengths; the RI was the product of the slenderness with the volume of fibers.
- Four empirical equations were developed to predict the toughness of concrete as a function of the RI. Equations (2) and (3) predicted the toughness of concrete reinforced with polypropylene fibers, measured at 6 and 4 mm TCOD. Pearson’s correlation coefficient is 0.68 and 0.69, respectively. Equations (4) and (5) predicted the residual strength, measured at 6 and 4 mm TCOD. Finally, these equations can be used for concrete with compressive strength of 40 to 45 MPa, slump ranging from 46 to 240 mm, elaborated with aggregates with a maximum nominal size of 25.4 mm.
- The coefficient of variation of the toughness calculated by the Barcelona method varies depending on the material and the pouring location. The COV of FRC with metallic fibers is 11.11% and 10.71% for mine and laboratory-made concrete, respectively. The COV of FRC with synthetic fibers is 18.81% and 15.05% for mine and laboratory-made concrete, respectively.

The limitation of this study lies in the method’s sensitivity to detect variations presented by the fibers in the structure. It is acknowledged that correlations among variables may present discrepancies compared to other tests. Investigating the sensitivity to capture variable variations for each method and comparing them is a future study that should be considered. Further studies could also involve varying the maximum nominal size of coarse aggregate and assessing its influence on post-cracking properties.

## Figures and Tables

**Table 1:** Design of concrete mixtures (kg/m^3^).

Type	PP Fibers 40 mm	PP Fibers 50 mm	PP Fibers 60 mm	Water	Cement	Fine Aggregate	Coarse Aggregate	Water Reducer
Pattern	-	-	-	226.6	502.8	721.1	891.2	7.1
D:0.4–40	3.6	-	-	226.6	502.8	721.1	891.2	7.1
D:0.4–50	-	3.6	-	226.6	502.8	721.1	891.2	7.1
D:0.4–60	-	-	3.6	226.6	502.8	721.1	891.2	7.1
D:0.8–40	7.2	-	-	226.6	502.8	721.1	891.2	7.1
D:0.8–50	-	7.2	-	226.6	502.8	721.1	891.2	7.1
D:0.8–60	-	-	7.2	226.6	502.8	721.1	891.2	7.1
D:1.2–40	10.8	-	-	226.6	502.8	721.1	891.2	7.1
D:1.2–50	-	10.8	-	226.6	502.8	721.1	891.2	7.1
D:1.2–60	-	-	10.8	226.6	502.8	721.1	891.2	7.1

**Table 2:** Characteristics of FRC in the fresh and hardened condition.

Batch	V_f (%)	l/d (-)	RI (V_f × l/d)	f_c (MPa)	COV (%)	f_t (MPa)	COV (%)	Slump (mm)
Pattern	0.0	0.0	0.0	42.2	4.7%	5.6	16.4%	240
D:0.4–40	0.4	46.5	18.6	41.2	3.0%	5.9	18.7%	210
D:0.4–50	0.4	58.1	23.3	42.9	4.3%	5.8	13.8%	210
D:0.4–60	0.4	69.8	27.9	39.6	2.6%	5.9	11.0%	140
D:0.8–40	0.8	46.5	37.2	39.6	4.1%	6.7	9.6%	175
D:0.8–50	0.8	58.1	46.5	41.2	7.4%	7.2	7.4%	70
D:0.8–60	0.8	69.8	55.8	42.9	3.6%	5.5	7.4%	125
D:1.2–40	1.2	46.5	55.8	42.6	1.2%	6.5	9.2%	95
D:1.2–50	1.2	58.1	69.8	41.0	8.6%	5.5	15.5%	46
D:1.2–60	1.2	69.8	83.7	43.6	18.8%	5.2	11.7%	111

**Table 3:** Flexural mechanical properties of concrete reinforced with polypropylene fibers using the Barcelona test.

Batch	RI (V_f × l/d)	Pmax (kN)	COV (%)	f_ct (MPa)	P_4 mm (kN)	COV (%)	f_res_4 mm (MPa)	T_4 mm (N × m)	COV (%)	P_6 mm (kN)	COV (%)	f_res_6 mm (MPa)	T_6 mm (N × m)	COV (%)
Pattern	0.0	128.3	8.1%	8.1	-	-	-	-	-	-	-	-	-	-
D:0.4–40	18.6	123.2	6.4%	7.7	24.9	14.6%	1.56	152.8	33.1%	18.6	16.6%	1.2	177.3	29.8%
D:0.4–50	23.3	134.2	4.1%	8.4	25.1	29.6%	1.58	174.6	17.4%	18.4	36.4%	1.2	221.3	19.1%
D:0.4–60	27.9	133.2	6.8%	8.4	29.8	24.1%	1.88	197.0	15.2%	16.3	48.7%	1.0	260.1	16.5%
D:0.8–40	37.2	133.3	4.0%	8.4	30.7	25.6%	1.93	200.5	16.2%	24.6	32.4%	1.5	257.8	18.9%
D:0.8–50	46.5	131.3	10.2%	8.3	38.0	13.0%	2.39	227.1	15.4%	29.1	10.3%	1.8	302.8	10.6%
D:0.8–60	55.8	121.2	6.8%	7.6	28.2	13.7%	1.77	186.0	11.4%	22.3	13.7%	1.4	235.9	11.2%
D:1.2–40	55.8	117.1	6.6%	7.4	35.3	13.1%	2.22	173.9	24.0%	27.4	15.7%	1.7	229.9	23.1%
D:1.2–50	69.8	126.6	4.8%	8.0	39.6	13.5%	2.49	226.9	10.6%	29.7	19.1%	1.9	295.9	11.7%
D:1.2–60	83.7	126.3	4.3%	7.9	38.9	9.8%	2.45	230.1	10.7%	29.1	25.9%	1.8	298.7	9.3%

**Table 4:** Pearson’s hypothesis for correlations between flexural properties and fiber variables.

Item	MV	IV	DV	R^2^	R	*p*	Null Hypothesis
1	l/d = 47	V_f	f_ct	18.96%	43.54%	0.565	no rejection
2	l/d = 58	V_f	f_ct	9.04%	30.07%	0.699	no rejection
3	l/d = 70	V_f	f_ct	21.75%	46.64%	0.534	no rejection
4	0.4	l/d	f_ct	67.25%	82.01%	0.388	no rejection
5	0.8	l/d	f_ct	86.95%	93.25%	0.235	no rejection
6	1.2	l/d	f_ct	5.41%	23.26%	0.767	no rejection
7	-	RI	f_ct	11.92%	34.53%	0.329	no rejection
8	l/d = 47	V_f	f_res_4 mm	84.03%	91.67%	0.083	rejected
9	l/d = 58	V_f	f_res_4 mm	86.30%	92.90%	0.071	rejected
10	l/d = 70	V_f	f_res_4 mm	77.90%	88.26%	0.117	no rejection
11	0.4	l/d	f_res_4 mm	78.83%	88.79%	0.304	no rejection
12	0.8	l/d	f_res_4 mm	6.18%	24.86%	0.840	no rejection
13	1.2	l/d	f_res_4 mm	60.41%	77.72%	0.433	no rejection
14	-	RI	f_res_4 mm	67.66%	82.26%	0.006	rejected
15	l/d = 47	V_f	f_res_6 mm	85.19%	92.30%	0.077	rejected
16	l/d = 58	V_f	f_res_6 mm	86.24%	92.87%	0.071	rejected
17	l/d = 70	V_f	f_res_6 mm	93.12%	96.50%	0.035	rejected
18	0.4	l/d	f_res_6 mm	75.21%	86.72%	0.332	no rejection
19	0.8	l/d	f_res_6 mm	5.89%	24.27%	0.844	no rejection
20	1.2	l/d	f_res_6 mm	24.79%	49.79%	0.668	no rejection
21	-	RI	f_res_6 mm	71.54%	84.58%	0.002	rejected
22	l/d = 47	V_f	T_4 mm	66.70%	81.67%	0.183	no rejection
23	l/d = 58	V_f	T_4 mm	77.34%	87.94%	0.121	no rejection
24	l/d = 70	V_f	T_4 mm	71.28%	84.43%	0.156	no rejection
25	0.4	l/d	T_4 mm	100.00%	100.00%	0.003	rejected
26	0.8	l/d	T_4 mm	12.25%	35.00%	0.772	no rejection
27	1.2	l/d	T_4 mm	65.89%	81.17%	0.397	no rejection
28	-	RI	T_4 mm	47.98%	69.27%	0.039	rejected
29	l/d = 47	V_f	T_6 mm	73.79%	85.90%	0.141	no rejection
30	l/d = 58	V_f	T_6 mm	78.12%	88.39%	0.116	no rejection
31	l/d = 70	V_f	T_6 mm	69.57%	83.41%	0.166	no rejection
32	0.4	l/d	T_6 mm	99.85%	99.92%	0.025	rejected
33	0.8	l/d	T_6 mm	10.46%	32.34%	0.790	no rejection
34	1.2	l/d	T_6 mm	77.84%	88.23%	0.312	no rejection
35	-	RI	T_6 mm	46.20%	67.97%	0.044	rejected

Note: MV = Moderate Variant, IV = Independent variant, DV = Dependent variant, R^2^ = Determination coefficient, R = Correlation coefficient, *p* = Significance value, l/d = Slenderness, V_f = Fiber volume, f_ct = Indirect tensile strength, f_res = Residual strength, RI = Reinforcement index, T = Toughness.

**Table 5:** Compilation of toughness results from other authors.

Item	Author	Material	Place	E_fib (GPa)	V_f (%)	L (mm)	d (mm)	l/d (-)	RI (V_f × l/d)	fres_4 mm (MPa)	COV (%)	T_4 mm (N × m)	COV (%)
1	[25]	steel	Laboratory	200	0.32	35	0.55	64	20	-	-	241.8	4.8%
		steel	Laboratory	200	0.64	35	0.55	64	41	-	-	350.6	6.2%
		steel	Laboratory	200	0.96	35	0.55	64	61	-	-	293.0	14.4%
2	[12]	steel	Laboratory	200	0.50	35	1.00	35	18	0.74	26.2%	186.6	14.4%
		steel	Laboratory	200	1.00	35	1.00	35	35	0.95	9.9%	235.6	5.6%
		steel	Laboratory	200	0.50	50	1.00	50	25	0.59	2.4%	193.6	8.5%
		steel	Laboratory	200	1.00	50	1.00	50	50	1.30	10.1%	326.8	5.7%
3	[26]	steel	Laboratory	210	0.38	35	0.55	64	25	1.09	19.2%	301.4	5.7%
		steel	Laboratory	210	0.38	42	0.61	69	26	0.90	21.0%	232.2	10.5%
		steel	Laboratory	210	0.38	49	0.72	68	26	0.99	8.8%	259.3	5.8%
		steel	Laboratory	210	0.38	60	0.84	72	27	1.14	19.3%	301.8	13.2%
4	[36]	syn	Laboratory	10	0.55	48	-	-	-	-	-	259.4	13.2%
		syn	Laboratory	10	0.99	48	-	-	-	-	-	265.8	11.6%
		steel	Laboratory	210	0.76	50	0.62	81	62	-	-	352.8	15.9%
		steel	Laboratory	210	0.51	50	0.62	81	41	-	-	310.6	2.2%
5	[19]	syn	Laboratory	-	0.55	48	0.91	53	29	-	-	230.0	13.6%
		syn	Laboratory	-	0.71	48	0.91	53	38	-	-	233.8	22.5%
		steel	Laboratory	200	0.32	50	0.75	67	21	-	-	236.0	13.1%
6	[11]	syn	Laboratory	7	0.46	54	0.87	62	29	0.35	11.4%	89.2	10.4%
		syn	Laboratory	7	0.46	54	0.87	62	29	0.22	18.2%	66.6	14.6%
		syn	Laboratory	7	0.46	54	0.87	62	29	0.34	50.0%	62.6	10.3%
		syn	Laboratory	7	0.46	54	0.87	62	29	0.34	17.6%	117.4	5.7%
		steel	Laboratory	210	0.32	35	0.54	65	21	0.49	30.6%	94.3	18.5%
		steel	Laboratory	210	0.32	35	0.54	65	21	0.59	30.5%	93.8	7.4%
		steel	Laboratory	210	0.32	35	0.54	65	21	0.58	36.2%	86.4	18.7%
		steel	Laboratory	210	0.32	35	0.54	65	21	0.57	7.0%	105.1	18.6%
		steel	Laboratory	210	0.51	35	0.54	65	33	0.82	17.1%	114.0	4.2%
		steel	Laboratory	210	0.76	35	0.54	65	50	0.91	17.6%	125.1	13.8%
7	[37]	steel	Laboratory	200	0.51	-	-	70	36	-	-	283.80	17.7%
8	[22]	syn	Tunnel	-	-	54	0.84	64	-	-	-	254.96	18.8%
		syn	Laboratory	-	0.44	54	0.84	64	28			175.0	-
		syn	Laboratory	-	0.88	54	0.84	64	57			230.0	-
		syn	Laboratory	-	1.32	54	0.84	64	85			260.0	-
9	[6]	steel	Tunnel	200	0.38	50	1.05	47	18	0.85	15.0%	248.2	10.8%
		steel	Tunnel	200	0.64	50	1.05	47	31	0.95	19.0%	277.7	11.6%
		steel	Tunnel	200	0.51	60	0.71	85	43	1.74	11.0%	391.2	6.8%
		steel	Tunnel	200	0.77	60	0.71	85	65	1.73	6.0%	389.3	8.3%
		steel	Tunnel	200	0.39	35	0.55	64	25	0.97	21.0%	314.0	6.0%
		steel	Tunnel	200	0.51	60	0.71	85	43	1.7	14.0%	433.6	9.7%
		steel	Tunnel	200	0.51	60	0.71	85	43	2.14	13.0%	461.3	8.6%
		steel	Tunnel	200	0.32	35	0.55	64	20	0.59	32.0%	176.9	19.8%
		steel	Tunnel	200	0.51	35	0.55	64	33	0.72	22.0%	198.3	18.4%

## Data Availability

The data supporting the findings of this study are available within the article.
